# NAPS: Integrating pose estimation and tag-based tracking

**DOI:** 10.1111/2041-210X.14201

**Published:** 2023-08-28

**Authors:** Scott W. Wolf, Dee M. Ruttenberg, Daniel Y. Knapp, Andrew E. Webb, Ian M. Traniello, Grace C. McKenzie-Smith, Sophie A. Leheny, Joshua W. Shaevitz, Sarah D. Kocher

**Affiliations:** 1 Lewis-Sigler Institute for Integrative Genomics, Princeton University, Princeton, New Jersey, USA; 2 Department of Physics, Princeton University, Princeton, New Jersey, USA; 3 Department of Ecology and Evolutionary Biology, Princeton University, Princeton, New Jersey, USA; 4 Department of Molecular Biology, Princeton University, Princeton, New Jersey, USA

**Keywords:** behavioural tracking, ethology, hybrid tracking, pose estimation, social networks, tag-based tracking

## Abstract

1. Significant advances in computational ethology have allowed the quantification of behaviour in unprecedented detail. Tracking animals in social groups, however, remains challenging as most existing methods can either capture pose or robustly retain individual identity over time but not both.

2. To capture finely resolved behaviours while maintaining individual identity, we built NAPS (NAPS is ArUco Plus SLEAP), a hybrid tracking framework that combines state-of-the-art, deep learning-based methods for pose estimation (SLEAP) with unique markers for identity persistence (ArUco). We show that this framework allows the exploration of the social dynamics of the common eastern bumblebee (*Bombus impatiens*).

3. We provide a stand-alone Python package for implementing this framework along with detailed documentation to allow for easy utilization and expansion. We show that NAPS can scale to long timescale experiments at a high frame rate and that it enables the investigation of detailed behavioural variation within individuals in a group.

4. Expanding the toolkit for capturing the constituent behaviours of social groups is essential for understanding the structure and dynamics of social networks. NAPS provides a key tool for capturing these behaviours and can provide critical data for understanding how individual variation influences collective dynamics.

## 1 | INTRODUCTION

**FIGURE 1:**
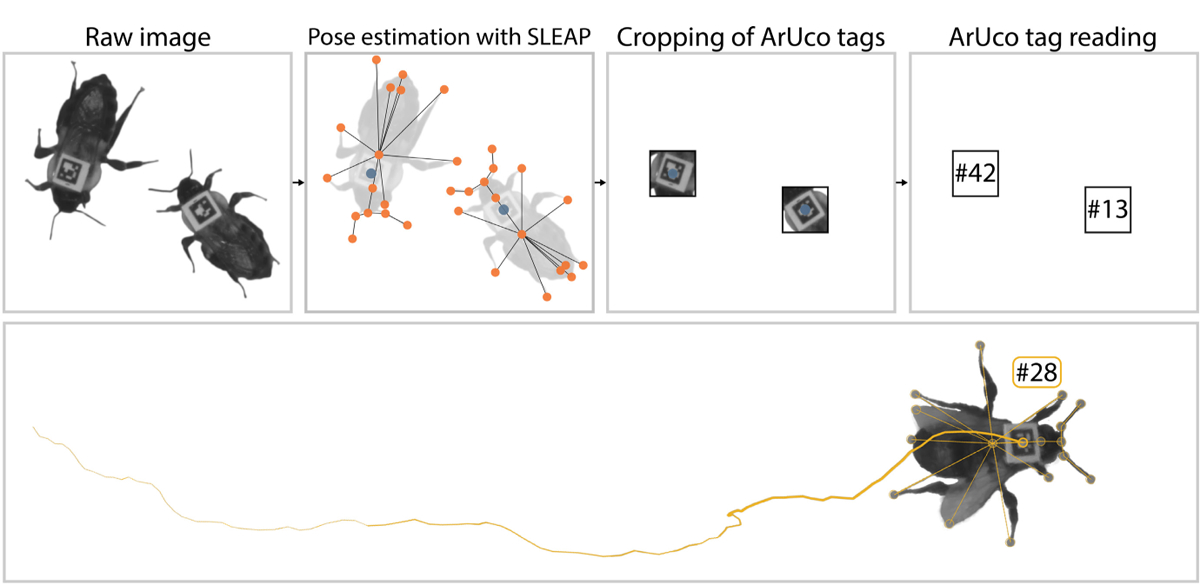
NAPS produces postural data with high-fidelity identity assignments from raw videos of tagged individuals. Top: Software pipeline. SLEAP is used to identify the location of each body part in the skeleton (orange dots) and the position of the ArUco tag (blue dot). NAPS crops the image around the tag and uses this as the input for ArUco tracking. Bottom: Tags are uniquely assigned to individual bees, and all instances from a single animal are combined to generate a unique identity. The orange line shows the tag’s location as the animal locomotes, increasing in width with time.

Massive advances in computing and imaging technologies enable new methods to study and quantify animal behaviour. Recently, many pose estimation tools, such as SLEAP and DeepLabCut (Lauer et al., 2022; Pereira et al., 2022), have been developed to track the location of individual body parts of multiple animals as nodes on a skeleton (Figure 1). With advancements in computational ethology, we are able to detect fine-grained behaviours, including grooming, touching and gait changes (Berman et al., 2014, 2016; Gernat et al., 2018; Geuther et al., 2021; Jones et al., 2020; Klibaite et al., 2022; Wang et al., 2022; Wiltschko et al., 2020). Despite these advances, maintaining the identity of individuals in multi-animal data (identity persistence) remains a challenge. This problem is especially pronounced when closely interacting animals may occlude each other’s body parts or when animals are partially or fully obscured by a complex environment. This problem is not always trivially solved with visual classifiers, as they struggle to classify morphologically similar individuals.

SLEAP, for example, provides two primary mechanisms for maintaining identity: temporal-based models, where identity is assigned based on features in previous frames, and appearance-based models, which use a neural network trained on known instances of a given individual to distinguish animals through differences in appearance. Temporal-based models encounter challenges due to physical occlusions, and these obstructions not only cause immediate problems but also lead to the propagation of errors over time. Appearance-based models require a significant time commitment for model training and struggle to distinguish visually similar individuals (Lauer et al., 2022; Pereira et al., 2022; Romero-Ferrero et al., 2019). Other methods, such as TRex and Argos, have relied on identifying where individuals are in extremely close proximity (crossing) and using neural networks specifically to maintain identity across these crosses (Sclocco et al., 2021; Walter & Couzin, 2021).

In parallel with deep learning methods for pose estimation, several unique identifier-based approaches (Alarcón-Nieto et al., 2018; Boenisch et al., 2018; Crall et al., 2015; Eagan et al., 2022; Gernat et al., 2018; Mersch et al., 2013) have been developed to maintain the identity of individuals. In these methods, a unique identifier affixed to an individual allows for tracking each individual’s position and orientation over time. While effectively addressing the identity persistence problem, many complex behaviours are not captured by these methods in isolation, as tag position only provides the position and orientation of a single point on the animal’s body.

Here, we introduce NAPS (NAPS is ArUco Plus SLEAP), a framework that combines the identity information from ArUco tags with temporal tracking and postural information from SLEAP (Figure 1). This innovative approach combines deep learning-based pose estimation with unique markers for each individual, enabling long-term pose tracking of individuals with robust identity assignment. This integrates and improves upon the identity persistence capabilities of other methods (Table S1). In the following sections, we document this framework, provide an example use case and discuss potential future applications.

## 2 | NAPS (NAPS IS ARUCO PLUS SLEAP)

NAPS uses a hybrid tracking algorithm to provide high-dimensional behavioural data compatible with recently developed computational ethology techniques. Similar hybrid tracking techniques have been introduced in the literature (Gal et al., 2020; Smith et al., 2022). The NAPS framework utilizes quantitative information about body part position and unique identifying markers to provide postural data with identity over long timescales and robust to complex interactions. We used SLEAP, a deep learning-based framework for multi-animal pose estimation, which has been previously shown to be robust in complex, naturalistic environments (Pereira et al., 2022; Wang et al., 2022). SLEAP is used to localize the positions of focal body parts and the ArUco tags from video data. These body parts localized by SLEAP form the nodes of a user-defined skeleton (Figure 1). The output of the SLEAP workflow is a set of predicted body part locations combined into skeletons and their corresponding identities. However, SLEAP will split tracks from a single animal into multiple tracks if it does not correctly assign identity and can also propagate track-switch errors. We used the identity information from ArUco tags to link these broken SLEAP tracks together and remedy track switches (Figure 1).

The first step in our framework uses SLEAP, which can localize body parts with or without an identifiable tag. The SLEAP skeleton must include the tag as a node but otherwise can have as many other nodes as desired (Figure 1). To enable identification with ArUco, the area around the tag node is cropped out for each individual, and each crop is processed with OpenCV’s ArUco detection module (Bradski, 2000; Garrido-Jurado et al., 2014). We assign an identity to each instance using the ArUco tags through the Kuhn–Munkres algorithm (Kuhn, 1955, 1956; Munkres, 1957). The ArUco tag ID is read for each instance, generating a binary matrix of tag ID and SLEAP instance coincidences, *I*_*i*,*j*_ (*t*) for frame *t*. To mitigate issues with misassigned and misread tags, we generate a cost matrix by subtracting these vectors in overlapping windows,

(1) $$C_{i,j}\left(t\right)=-\overset{t+w}{\underset{k=t-w}{\sum }}I_{i,j}\left(k\right).$$

For the results shown here, we use a sliding window size of 41 frames (*w* = 20). We assign IDs to each instance by finding the minimum of the cost function using the Kuhn–Munkres algorithm as implemented in SciPy (Virtanen et al., 2020),

(2) $$\underset{i\in \text{ Tracks}}{\sum }\underset{j\in \text{ Tags}}{\sum }C_{i,j}\cdot A_{i,j},$$

where *A* is a permutation matrix. Selecting larger window sizes may cause identity switches to propagate further before correction and shorter windows are less robust to misidentifications. We find qualitatively that this has little effect on our data set since few swaps occur in the simple arena. In practice, users may need to adjust the window size depending on their experimental design and the resulting frequency of ArUco tag identifications as well as the behaviours of interest (for further explanation, Supplementary Information).

**FIGURE 2:**
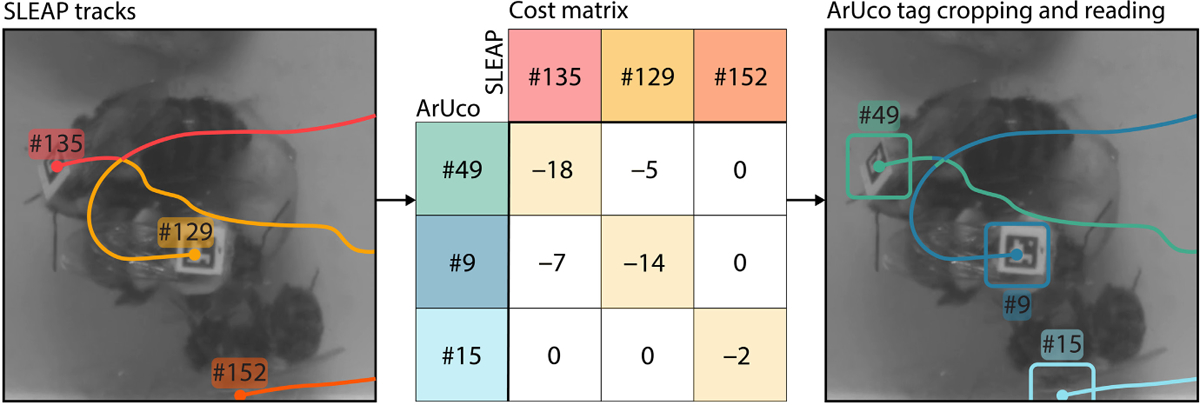
Left: Raw images of three interacting bees. Overlaid in colour are the SLEAP-identified tracks that show a track-switch error. Middle: ArUco-based cost matrix, *C*, for this frame. Right: NAPS-corrected tracks overlaid without a track-switch error. The rounded boxes represent the area cropped out and subsequently read with OpenCV’s ArUco detection module for each instance.

Here, we only rely directly on the SLEAP-based identity assignment, that is forward propagation of temporal tracks, for instances where the ArUco tag was read for the track previously and the ArUco-based ID was not reassigned to another track. While the initial, temporal-model based tracks may also have spurious assignments, and these may propagate through NAPS, we have found that they are rare and easy to correct in postprocessing. This matching framework allows us to fix track switches and other identity errors produced by temporal models (Figure 2).

The result is an easy-to-use system for combining the high-dimensional postural data from SLEAP with high-fidelity identity assignment from ArUco tags. This framework is trivially parallelizable, allowing for easy scaling to large data sets consisting of millions of images or more. Such parallelization can be achieved by either processing multiple videos simultaneously on separate computational units or by dividing a single lengthy video into smaller segments and analysing them independently. This adaptability ensures efficient data handling, even for extensive and high-resolution video data sets.

## 3 | EXAMPLE USAGE AND ASSESSMENT

As validation of our framework, we ran NAPS on an example data set consisting of three 1-h video segments of 50 bumblebees (*Bombus impatiens*), including a single queen in a 235 mm × 235 mm arena (Figures S1 and S2). Each hour segment was pulled from a 24-h recording, with videos starting at 04:00, 12:00 and 20:00. The 50-bee colony was constructed from randomly sampled individuals, except the queen, from a colony obtained from Koppert Biological Systems (August 2022) containing approximately 200 workers and a single queen. We printed 4.25 mm 5 × 5 ArUco tags generated from the 5X5_50 set on TerraSlate 5 Mil paper (TerraSlate) and cut these to 4.25 × 4.25 mm using a Silhouette Cameo cutting machine (Silhouette). Before tagging, bees were sedated via cooling on wet ice, and an ArUco tag was affixed to the dorsal side of the thorax using cyanoacrylate glue (Loctite) (Figure 1). Cyanoacrylate glue does not have adverse affects on behaviour or mortality in bees (Gernat et al., 2018) and previous work only observed an acute increase in grooming post-tagging that rapidly dissipated as bees acclimated (Crall et al., 2015). As each tag was placed, we ensured that it could be read using a real-time ArUco tag reader and noted the tag number.

Following tagging, individuals were placed in arenas made from laser-cut acrylic and 3D-printed polylactic acid. Cotton wicks soaking in sugar water were placed in one corner of the arena to allow ad libitum feeding, and pollen mixed with honey was added for nutrition. The addition of these elements increases the complexity of the tracking environment and allows us to test NAPS’ performance under experimental conditions where its utility is most relevant.

Arenas were covered with clear acrylic and lit using two high-intensity 850 nm LED light bars (Smart Vision Lights L300 Linear Light Bar) to allow continuous imaging of the bees while preserving a hive-like visual environment, as bees are unable to see infrared light (Figure S1). We imaged the arenas from above using a Basler acA5472–17um (Basler AG) camera recording 3664px × 3664px frames at 20 frames per second. Recordings were taken using a modified version of campy, a Python package developed for real-time video compression, to drastically reduce file sizes (Severson, 2021). The videos have a spatial resolution of 15.5 pixels/mm, allowing us to capture fine-grained behaviours. Because each bumblebee worker varies between approximately 9 and 14 mm in length, the resulting pixel length of each worker is approximately between 139.5px and 217px in the recordings (Williams et al., 2014).

After video acquisition, we processed each video with SLEAP. We used a 17-node skeleton marking the head, two thorax points, abdomen, left and right antennal joints, left and right antennae, left and right wings, the pretarsus of each leg and the ArUco tag. We trained on 31 frames sampled across the three videos. The 31 frames in our training set provided 1550 labelled instances encompassing the 50 individuals, and the resulting model is highly accurate (Figure S3). We performed inference using Nvidia A100 GPUs to generate initial tracks with temporal association. For this step, we use matching based on Intersection over Union (IoU) similarity with a 5-frame window. We also used SLEAP’s pre-and postculling arguments, target instance count and single track break connecting flag. The complete SLEAP command is provided in the Git repository. In running NAPS on this data set, we first identified an ideal set of parameters for the data, noting the approximate size that each crop needed to be around the tag node, selecting a window size (20 frames on each side of the focal frame) that allows robust identification and yet quick correction, and adjusted the OpenCV ArUco detector parameters to fit the data set. This resulting parameters were the NAPS defaults except for the following parameters: tag node name (‘tag’), ArUco tag set (DICT_5X5_50), half rolling window size (20 frames) and crop size (100px). More information for resolving ideal parameters can be found at naps.rtfd.io/en/latest/cli. html. Following identifying these parameters, we ran NAPS on the data set. The exact command utilized for this data set is included in the data repository.

**FIGURE 3:**
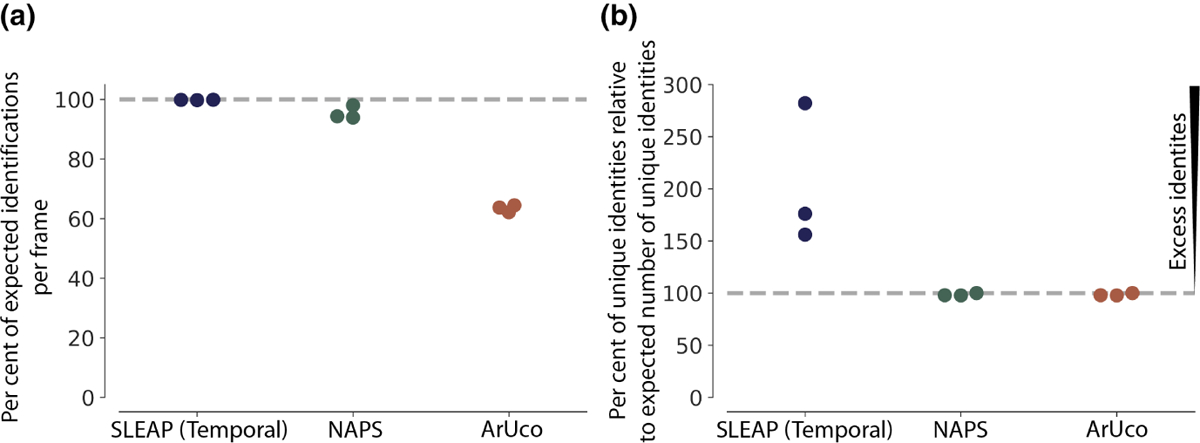
(a) A comparison of the per cent of expected identifications per frame by tracking method (SLEAP, NAPS and ArUco) inour example data set. For our data set, we expect all 50 bees to be identified by SLEAP. For NAPS and ArUco, we expect that all active individuals, where the ArUco tag should be detected at some point throughout the video, to be detected. This number varies from 44 to 48 depending on the video. Here, we see that NAPS captures a similar percentage of expected individuals per frame compared with SLEAP and significantly more than ArUco alone. We also validated the resulting tracks by hand-validating tracks with identity assignments. We randomly sampled three frames from each of our videos (nine total, 409 identities), and found a 100% match between the identity assigned by NAPS and the true identity. (b) A comparison of the per cent of the expected number of unique identities relative to the expected number of unique identities per video. We expect 50 unique identities for SLEAP and from 44 to 48 for NAPS and ArUco based on the number of active individuals in each video. SLEAP produces significantly more identities than expected while NAPS and ArUco generate almost exactly as many identities as expected.

Finally, to compare the results from SLEAP, NAPS and ArUco on our example data set, we calculated both the per cent of expected identifications (instances) per frame (Figure 3a) and the per cent of realized identities (i.e. unique identities found) per video relative to the number of expected identities (Figure 3b). The number of expected instances identified in each frame varies by method, with SLEAP expected to identify all instances even when individuals are inverted or not active. ArUco can only capture instances in a given frame when the tag is identifiable, and the number of expected instances for this method is the number of bees which appeared active. NAPS will have the same number of expected instances per frame as ArUco but is able to capture instances detected by SLEAP but missed by ArUco in a given frame. We find that SLEAP is able to detect nearly all expected instances in each frame, NAPS captures almost as many instances as expected, and ArUco captures ~63% of the expected number of instances per frame (Figure 3a). We also validated the resulting NAPS identity assignment by hand. We randomly sampled three frames from each of our videos (nine frames, 409 identity assignments) and compared the NAPS assigned identity with the hand-annotated identity and found 100% accuracy. Of the individuals assigned an identity, all were correctly assigned.

The relative per cent of expected identities assigned throughout a video also varies across methods, with SLEAP expected to detect all visible bees in a video, and NAPS and ArUco are only expected to detect bees that were active. SLEAP, using temporal tracking, assigns far more identities than expected, between ~156% and ~286% of the expected number (Figure 3b). NAPS and ArUco, with identities regulated by the detection of each ArUco tag, capture almost exactly the expected number of identities. These two metrics show that, while SLEAP is sufficient for identifying instances at the frame level and ArUco is sufficient for identifying the correct number of instances, NAPS enables the identification of the correct number of instances at the frame level while maintaining the correct number of identities across the video. Furthermore, even within stretches of frames where equal numbers of identities exist, temporal identity tracking may cause identity swaps. In this case, NAPS will correct track switches to avoid the propagation of errors as shown in Figure 2.

Using this data set, we also benchmarked the speed of NAPS relative to SLEAP alone and ArUco-based tracking alone. Since SLEAP and NAPS utilize a GPU while ArUco only uses CPU, hardware requirements vary between the methods. We utilized a compute node with eight cores (AMD EPYC) and 64G of RAM. For SLEAP, where a GPU significantly increases speed, a Nvidia A100 GPU was also used. With this hardware, SLEAP ran at approximately 3.6 frames per second, resulting in a complete run for a1 h video (72,000 frames) taking approximately 5.5 h. The additional NAPS step took approximately 40 min to run. ArUco detection alone ran slightly slower than real-time, varying between 65 and 75 min for a single hour of video.

**FIGURE 4:**
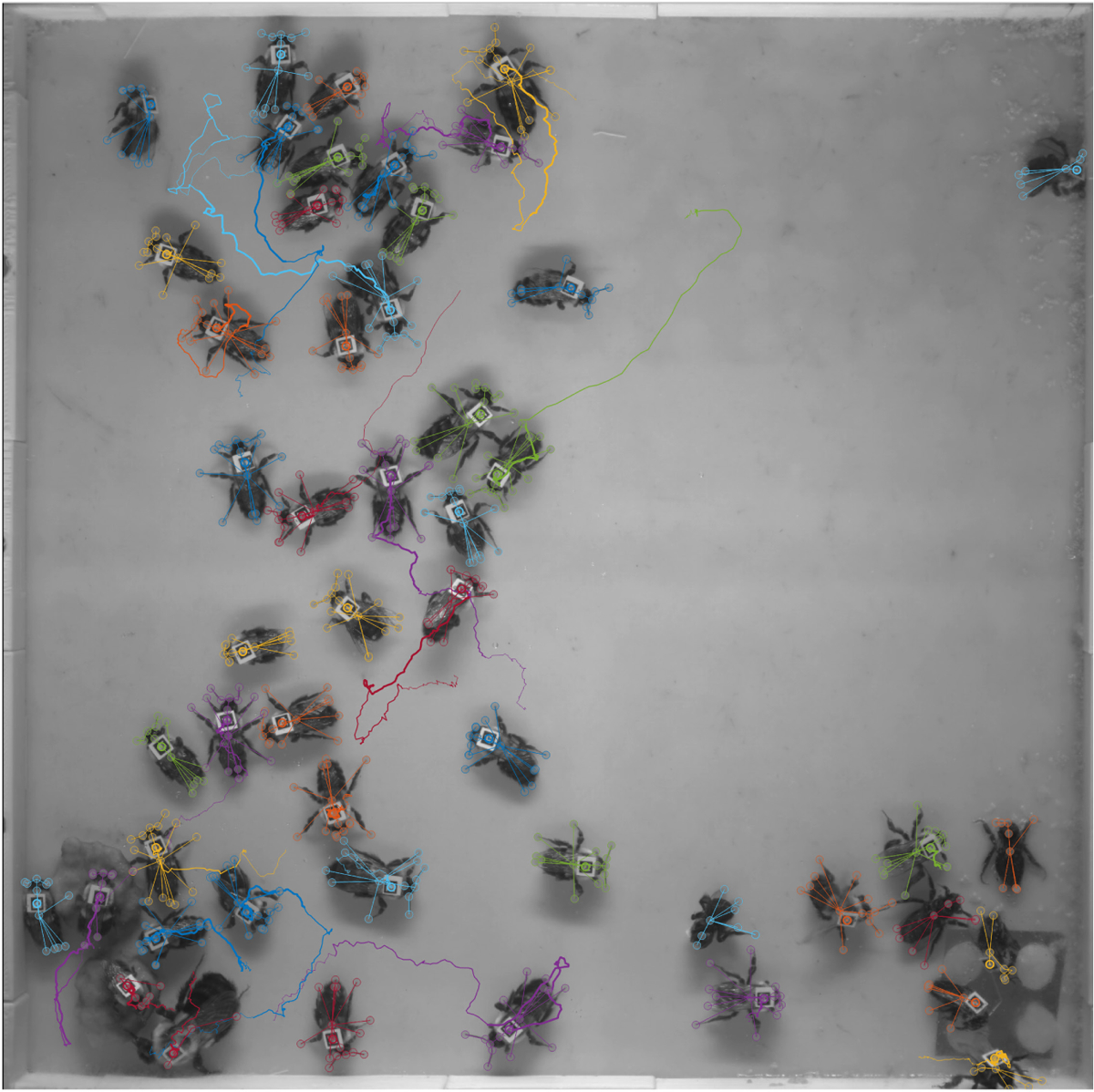
Example of a single frame from the output of NAPS. Traces for each individual, by colour, show the animal’s location in the previous 250 frames (12.5 s). NAPS produces robust identity persistence and highly accurate localization of nodes for individuals. Here, all tracks are included in this image, including those not assigned to an ArUco tag.

**FIGURE 5:**
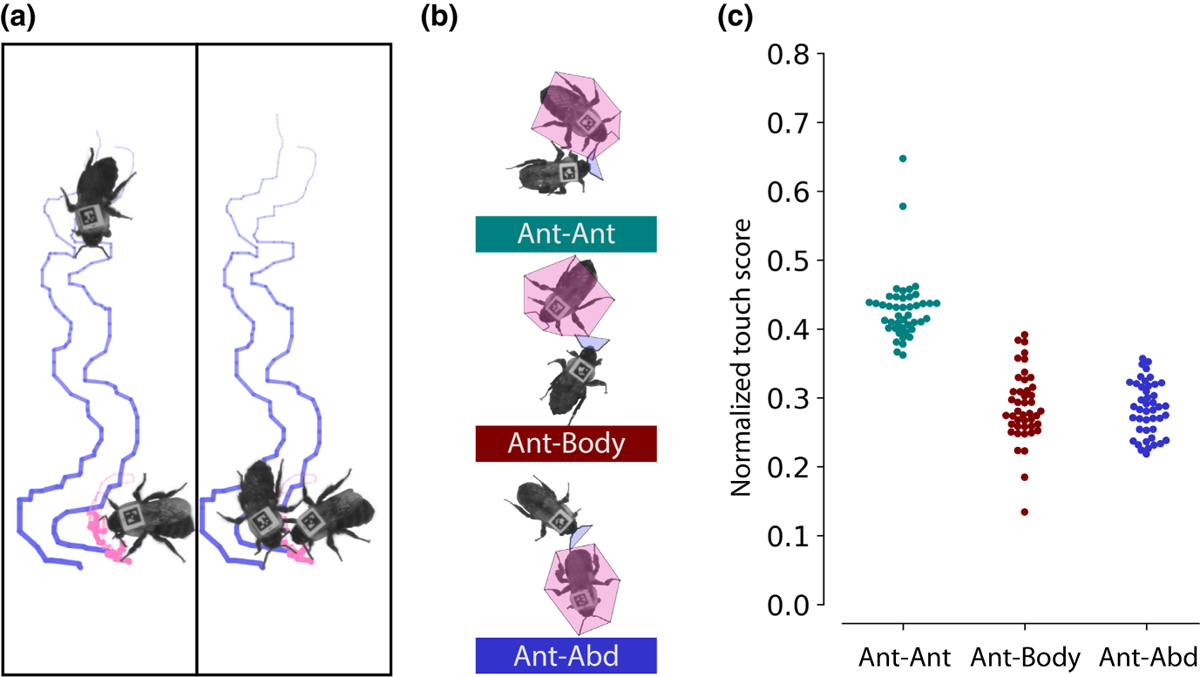
(a) Antennal tip traces of two bees during a brief interaction. The SLEAP coordinates of the antennal tips of two bees are plotted for 250 frames (12.5 s), providing spatiotemporal information about a single social interaction. (b) Schematic of antennal touch identifier (Wang et al., 2022). Instances of overlap between the antennal zone of the focal bee and the antennal (ant), body, or abdominal (abd) edges of the convex hull target bee are counted as touches. A bee can touch more than one of these zones at any moment. (c) Plot of antennal touches of the focal bee (blue) to three target bees (pink). Normalized touch scores across all bees with unique identities in all three sample videos (*n* = 43 workers, one queen). For each touch category, the proportion of all touches for a given target bee, averaged across each video, is normalized to account for the different sizes of each body region and plotted by touch category (Wang et al., 2022).

The behavioural data from NAPS allow us to carry out detailed behaviour analyses across dense groups (Figure 4). For example, since bumblebees use their antennae for social interaction and communication, we labelled them as nodes in our SLEAP skeleton. This allows us to track antennal interactions across all pairs of bees in our example data set (Figure 5a). Following the previous analysis from Wang et al. (2022), we quantified the amount of time each individual spent in different types of social interactions using a direct, convex-hull-based touch detector (Figure 5b). Bumblebees can gain access to different chemical cues by antennating on different locations, and we can observe the time series of different modes of antennation (to the antennae, body or abdomen) for a focal bee interacting with other bees in the arena. By normalizing these interactions by their likelihood (Wang et al., 2022), we find that the antennae–antennae mode of touching is preferred in this large group of 50 interacting bees (Figure 5c). This granular measure of an important behaviour demonstrates how NAPS can extend the capacity of behavioural tracking platforms by quantifying previously difficult-to-measure behaviours of individuals in dense groups far larger than previously accessible.

## 4 | DISCUSSION

Past studies of social behaviours utilized fine-grained behavioural data or persistent identity tracking with coarse behavioural tracking. NAPS combines these two types of data by integrating the pose estimation capabilities of SLEAP with the high-fidelity identity persistence of ArUco, enabling the concurrent analysis of behaviour at both the individual and collective levels. This provides new opportunities to examine how individual behaviours vary in social contexts and the role of individual variation in shaping group and collective dynamics. Within a group, even genetically identical individuals may differ in their behaviours (Bierbach et al., 2017; Herbert-Read et al., 2013; Landeau & Terborgh, 1986; Werkhoven et al., 2021). With NAPS, users can probe how individual variation affects the dynamics of collective decision-making (Cook et al., 2020), the establishment of leadership and dominance (Pettit et al., 2015; Zanette & Field, 2009), how individuals adjust their behaviours in response to their local environment (Conradt & Roper, 2005) and the structure of individual and collective dynamics and their evolution (Hernández et al., 2021).

## Supplementary Material

Supplementary Information

## Data Availability

NAPS is an open-source framework. The source code is available on GitHub at github.com/kocherlab/naps and it is archived at https://doi.org/10.5281/zenodo.7023698. The data set used in this manuscript is available on Princeton University’s DataSpace (https://doi.org/10.34770/6t6b-9545). To facilitate the broader usage of NAPS, we provide documentation, hosted at naps.rtfd.io, along with tutorials and example Jupyter notebooks (Brandl, 2022). The specific command line parameters for NAPS, in addition to documentation on the default values, can be found at naps.rtfd.io/en/latest/cli.html. We have released NAPS on Anaconda and PyPI, tested NAPS to nearly complete coverage (>94%), and new features are under continuous development. Furthermore, we illustrate the usage of NAPS through Google Colaboratory notebooks showing that the resources for a complete workflow, including SLEAP, are easily accessible using only free cloud computing resources. While the NAPS framework relies on ArUco tags for individual identification, it can be directly extended to use any other type of visually distinguishable marker, such as colour tags. This enables many different applications, including experiments where direct tagging may not be plausible. In parallel, NAPS users can take advantage of the flexibility of SLEAP, including the ability of end users to specify the nodes to track and capture previously elusive behaviours.
