# Supplementary material for: NAPS: Integrating pose estimation and tag-based tracking
Source: Methods Ecol Evol. Author manuscript; Available in PMC 2024 Apr 26. (PMC11052584; doi:10.1111/2041-210X.14201)
Supplement: Supplementary Information [file NIHMS1960171-supplement-Supplementary_Information.pdf]

Supplementary Information

Supplementary Note: Fidelity and Responsiveness to Temporal Window Size Adjustment

Choosing an optimal window size in a matching algorithm is a key decision that influences the trade-off between fidelity (the ability to maintain accurate tracking of identities) and responsiveness (the ability to quickly respond to identity swaps). This is particularly relevant in the context of tracking multiple individuals in a video, where identity swaps and other confusions can occur, such as tracking bees in a colony. We use the Kuhn-Munkres algorithm for assigning identities based on detected ArUco tags on the individuals. In ideal conditions, correct assignments would be represented by the diagonal of the cost matrices $C_{i,j}(t)$ shown below having the lowest values, corresponding to correctly assigned identities. However, the ArUco tag identification is not always perfect. SLEAP may occasionally assign nodes to incorrect tracks, and tags can be misread or not read at all in frames due to occlusions or other issues. Therefore, the choice of window size can have substantial effects on the resulting assignments and their accuracy.

In our framework, we construct a cost matrix $C_{i,j}(t)$ from the binary matrix of ArUco tag and SLEAP instance coincidences, $I_{i,j}(t)$ for each frame t. Each element of the cost matrix is computed as the negative summation of the coincidences over an overlapping window of size $2w+1$ centered on the frame:

$$C_{i,j}(t) = -\sum_{k=t-w}^{t+w} I_{i,j}(k) \tag{S1}$$

We assign IDs to each instance by finding the minimum of the cost function using the Kuhn-Munkres algorithm as implemented in SciPy, represented by the following equation where A is a permutation matrix:

$$\sum_{i \in \text{Tracks}} \sum_{j \in \text{Tags}} C_{i,j} \cdot A_{i,j} \tag{S2}$$

Consider three different window sizes and their impact on the matching process:

Small window size (3 frames; w=1). With a small window size, the algorithm can respond quickly to identity swaps, but it may also lead to an increase in incorrect assignments due to short-term ambiguities in tag readings. The cost matrix obtained may look like:

$$C_{i,j}(t) = \begin{bmatrix} -2 & -1 & 0 \\ 0 & -1 & -2 \\ 0 & -2 & -1 \end{bmatrix}$$

Here, due to the small window size, the first instance is correctly assigned to the first tag, but the second instance is incorrectly assigned to the third tag and the third instance to the second tag. This results in a deviation from the ideal diagonal alignment and shows how small windows can lead to misassignments.

Medium window size (41 frames; w=20). Increasing the window size helps to mitigate the impact of missed tag readings but also delays the algorithm's response to true identity swaps. The cost matrix obtained may look like:

$$C_{i,j}(t) = \begin{bmatrix} -20 & -3 & -2 \\ -3 & -21 & -2 \\ -2 & -3 & -20 \end{bmatrix}$$

Despite the missed tag readings, the larger window size allows for correct assignments as the correct identifications outweigh the missed tag identifications over the window of frames.

Large window size (101 frames; w=50). With a large window size, the algorithm shows high fidelity in identity assignments, even in the case of numerous missed tag readings. However, it can be slow to respond to rapid identity swaps. The cost matrix obtained may look like:

$$C_{i,j}(t) = \begin{bmatrix} -50 & -3 & -8 \\ -3 & -51 & -1 \\ -7 & -3 & -51 \end{bmatrix}$$

Despite a substantial number of incorrectly attributed tag readings, the large window size ensures correct assignments as the correct identifications far outweigh the missed identifications over the large window of frames.

However, identity swaps would take a relatively long period of time to correct, and in the case of tracks that swap twice in this window, can be completely missed.
These examples illustrate the need to carefully choose the window size based on the specifics of the experimental design and the nature of the data.

Tables

Software	Multi-animal Tracking	Direct CNN-based Identification	Marker-based Identification	Pose Estimation	Limitations	Citation
NAPS	✓		✓	✓	Requires individuals to be marked with ArUco tags. SLEAP needs to be run prior to running NAPS.	This manuscript
Argos	✓				Requires individuals to be separable from the background. Propagates identity errors over time.	Ray & Stopfer 2022
idtracker.ai	✓	✓			Large training overhead. Requires individuals to be easily separable from the background.	Romero-Ferrero et al., 2019
TRex	✓	✓			Requires individuals to be easily segmentable from the background.	Walter & Couzin 2021
AlphaTracker	✓			✓	Optimized for mice and relies on CNNs for dealing with track crosses. Requires individuals to be easily segmentable from the background.	Chen et al., 2023
SLEAP	✓	✓		✓	Large training overhead for appearance-based models. Temporal-models for for identification propagates identity swaps and cannot re-identify individuals who leave and reenter the frame.	Pereira et al., 2022
DeepLabCut	✓	✓		✓	Large training overhead for appearance-based models. Temporal-models for for identification propagates identity swaps and cannot re-identify individuals who leave and reenter the frame.	Lauer et al., 2022
anTraX	✓		✓		Color tagging is required.	Gal et al., 2020
ArUco, BeeTag, . . .	✓		✓		Tracking is lost when the markers are not in view.	Alarcón-Nieto et al., 2018 and Crall et al., 2018 for example

Table S1. This table presents a comparison of multi-animal tracking software. The "Direct CNN-based Identification" column denotes software's use of a trained Convolutional Neural Network (CNN) to directly identify individuals. The "Marker-based Identification" column indicates the software's reliance on external markers for identifying animals. "Pose Estimation" represents a software's ability to predict the pose of an animals' body parts beyond a single point or blob features. NAPS is unique among these in that it uses marker-based identification and provides postural data.

Figures

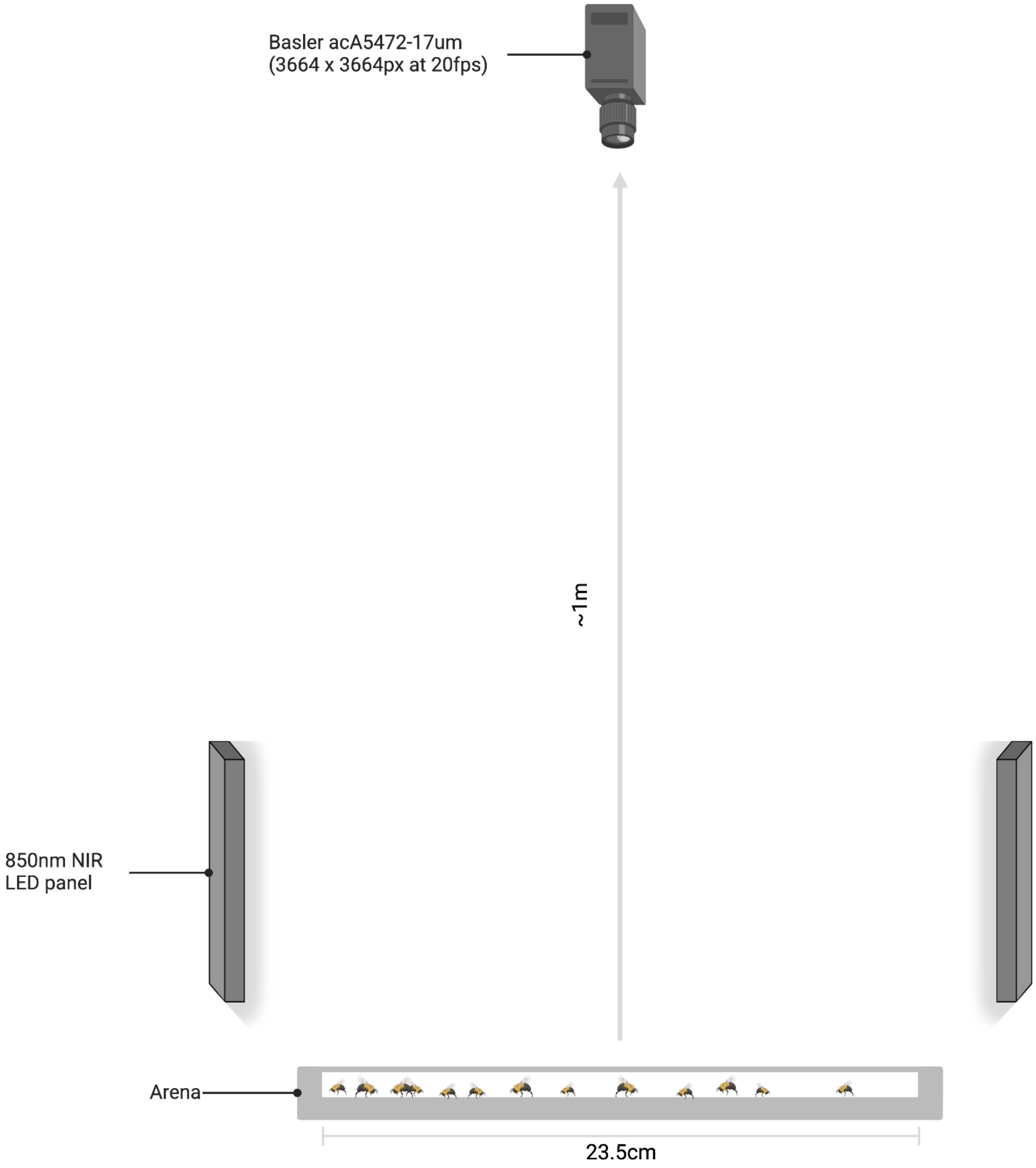

Figure S1. Diagram of the imaging setup used for capturing the example bumblebee data used. A camera facing directly down on the custom arena is flanked with two 850nm infrared LED light bars to allow continuous imaging of the bees without perturbation, as bees are unable to see infrared light. The resulting imaging setup allows high-quality imaging of large groups of individuals in a hive-like environment.

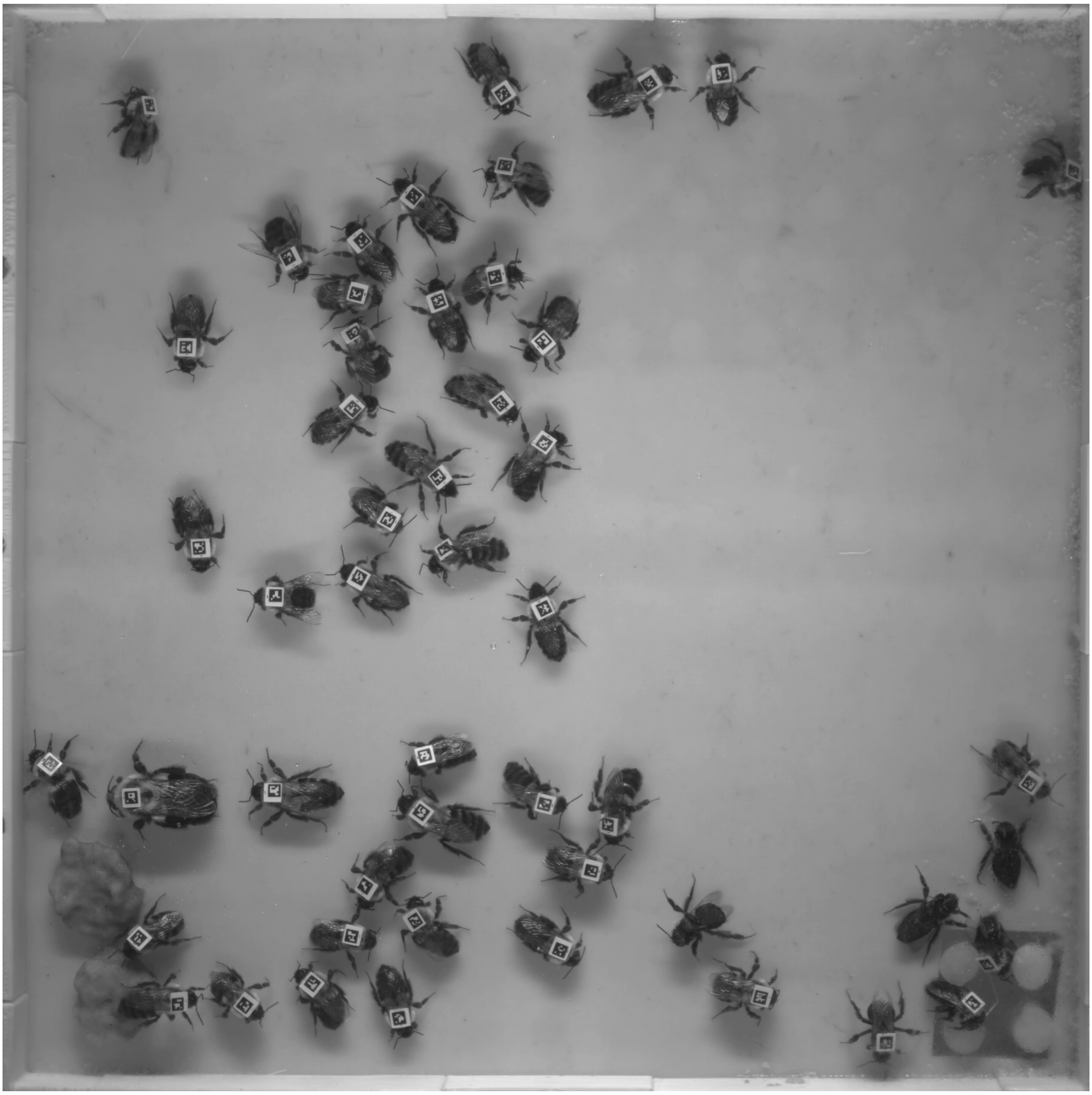

Figure S2. Example image pulled from the data set. Here, we see the density of individuals and the complex environment introduced by pollen dough (lower left) and sugar-water wicks for feeding (lower right).

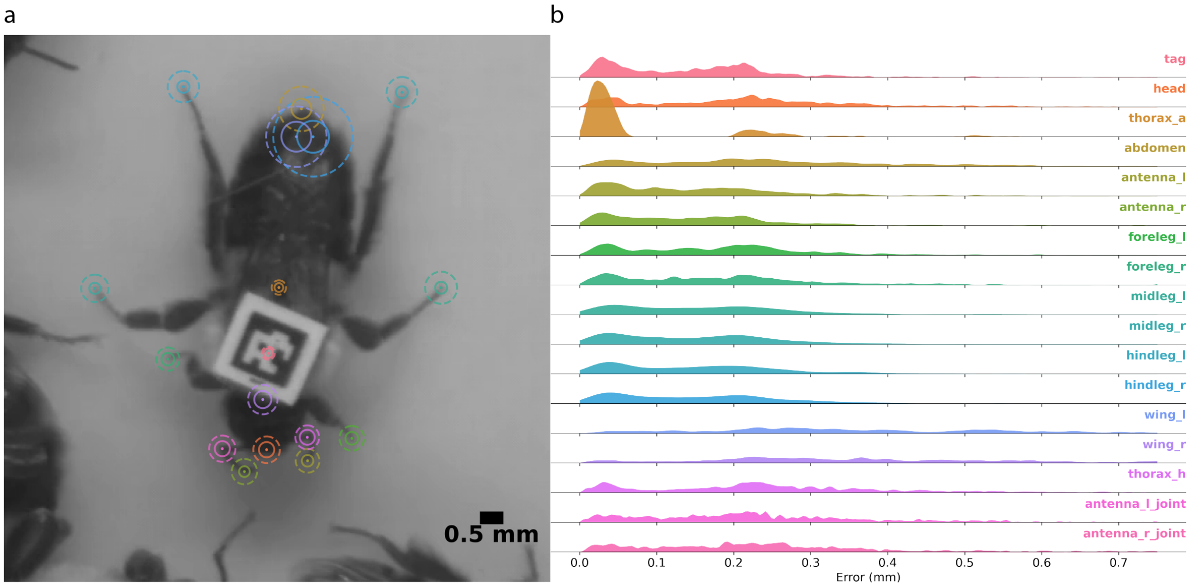

Figure S3. Node-wise localization accuracy. **a** shows the 75th percentile (solid) and 90th percentile (dashed) error for held-out validation set. **b** shows the distribution of localization errors on the same validation set. This distribution is clipped to (0, 0.75) for visibility.
